# Protamine-Based Strategies for RNA Transfection

**DOI:** 10.3390/pharmaceutics13060877

**Published:** 2021-06-14

**Authors:** Natalia Teresa Jarzebska, Mark Mellett, Julia Frei, Thomas M. Kündig, Steve Pascolo

**Affiliations:** 1 Department of Dermatology, University Hospital Zürich (USZ), University of Zürich (UZH), Raemistrasse 100, 8091 Zürich, Switzerland; NataliaTeresa.Jarzebska@usz.ch (N.T.J.); Mark.Mellett@usz.ch (M.M.); Julia.Frei@usz.ch (J.F.); Thomas.Kuendig@usz.ch (T.M.K.); 2 Faculty of Science, University of Zürich, 8091 Zürich, Switzerland; 3 Faculty of Medicine, University of Zürich, 8091 Zürich, Switzerland

**Keywords:** RNA, protamine, transfection, cancer therapy, vaccines

## Abstract

Protamine is a natural cationic peptide mixture mostly known as a drug for the neutralization of heparin and as a compound in formulations of slow-release insulin. Protamine is also used for cellular delivery of nucleic acids due to opposite charge-driven coupling. This year marks 60 years since the first use of Protamine as a transfection enhancement agent. Since then, Protamine has been broadly used as a stabilization agent for RNA delivery. It has also been involved in several compositions for RNA-based vaccinations in clinical development. Protamine stabilization of RNA shows double functionality: it not only protects RNA from degradation within biological systems, but also enhances penetration into cells. A Protamine-based RNA delivery system is a flexible and versatile platform that can be adjusted according to therapeutic goals: fused with targeting antibodies for precise delivery, digested into a cell penetrating peptide for better transfection efficiency or not-covalently mixed with functional polymers. This manuscript gives an overview of the strategies employed in protamine-based RNA delivery, including the optimization of the nucleic acid’s stability and translational efficiency, as well as the regulation of its immunostimulatory properties from early studies to recent developments.

## 1. Background

### 1.1. The Early Work

Friedrich Miescher started the first known studies on nucleoproteins like Protamine in the 1870s. It was then when he first identified two principal components of salmon spermatozoa, in addition to the acidic nuclein (DNA) he found an alkaline protein for which he coined the term ‘Protamin’ [1,2].

**Figure 1:**
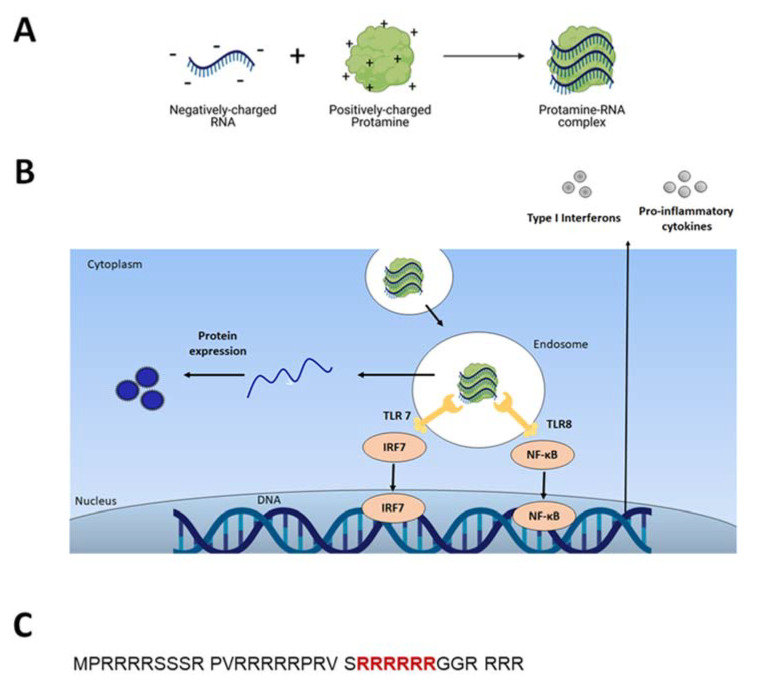
(**A**) Positively charged Protamine spontaneously assembles with negatively charged nucleic acids (here mRNA), formulating nanocomplexes; (**B**) The Protamine-RNA complex is internalized into the cell via endosomes. RNA acts as danger signals that trigger TLR7/8 to stimulate innate immune responses (see paragraph 3). mRNA released into the cytoplasm is translated into the desired protein (see paragraph 2); (**C**) Amino acid sequence of salmon sperm-derived protamine. Nuclear localization signal (NLS) highlighted in red [13].

Protamine is a naturally occurring protein containing more than two-thirds of positively charged L-arginine and is known to condense DNA during spermatogenesis. Due to the high amount of cationic L-arginine, protamine has the ability to complex nucleic acids (DNA and RNAs) and protect them from enzymatic degradation in biological systems (Figure 1A) [3].

60 years ago, Harold Amos, published the first report on the use of Protamine as an RNA carrier for uptake by eukaryotic cells [4]. He observed that the addition of Protamine sulfate to cultured chick embryo cells protected RNA from degradation. In his experiments, Protamine enhanced RNA uptake by 8 to 20 times more, in comparison with addition of naked mRNA. The same year, Christine Smull and colleagues confirmed Amos’ discovery, when they found that the addition of Protamine sulfate to cell culture can increase the infectivity of poliovirus RNA [5].

### 1.2. Protamine-RNA Complexes Characterization

Due to its cationic nature, Protamine spontaneously associates with purified, recombinant or chemically synthesized nucleic acids and forms complexes of up to several hundred nanometers in diameter (Figure 1A), [3,6,7]. The mechanism of Protamine binding to RNA was elucidated by R. Wade Warrant in 1978 [8]. In the presence of nucleic acids, Protamine molecules change their conformation from a random coil structure to a structure containing one or more alpha-helical segments. Protamine molecules bind to RNA nonspecifically, as in the study Protamine bound to all available parts of transfer-RNA (tRNA). The stoichiometry of positive charges of protamine to negative charges of nucleic acid was established to be approximately 1:1 [8].

Several groups have refined the formulation of Protamine-RNA particles and have identified conditions that allow for the production of homogenous nanoparticles upon mixing Protamine and RNA [9]. The average size of the particles can be precisely defined according to: (i) the salt concentration in the solutions used to dilute Protamine and RNA, (ii) the ratio of Protamine to RNA and (iii) the concentration of Protamine and RNA [10]. Thus, it is possible to generate particles with an average diameter from specifically 50 nanometer (nm) up to 1000 nm, depending on the needs.

### 1.3. Transfection Enhancer

Protamine can facilitate cell transfection as arginine-rich motives appear in viral translocation sequences. Indeed, Reynolds et al. [11] in their studies with rhodamine-modified protamine, observed that Protamine has membrane-translocating activity comparable to that of the HIV TAT peptide. Both compounds, Protamine and TAT peptide, showed strong nuclear localization and similar dependence on time and concentration: the complete internalization of both peptides was complete after 1 h post addition to the cell culture. Nuclear targeting of Protamine complexes was described in detail in the work of Vighi and colleagues in their studies on solid lipid nanoparticles containing Protamine [12]. Six consecutive arginines are postulated to be the nuclear localization signal (Figure 1C) [13]. Such specific intracellular localization should not be surprising regarding the fact that Protamine’s primary biological function is replacing histones during spermatogenesis [14].

## 2. Protamine-Based Particles

When delivering nucleic acids for therapeutic purposes, several obstacles for the exogenous molecules have to be overcome [15,16]. Among them, barriers of entry into the cell and cytoplasm present perhaps the most difficult challenge [17]. As nucleic acids are large, hydrophilic and anionic molecules, they do not readily traverse the hydrophobic lipid bilayer of the cell [18].

The use of Protamine as transfection agent arose from the need to improve in vitro and in vivo gene transfer efficiency by enhancing transfection efficiency based on condensing DNA or RNA in a manner similar to natural vectors such as virus or sperm [19,20]. Such virus-like structures would potentially have facilitated entry into the cell (Figure 1B) [21].

### 2.1. Protamine-Based Polyplexes

Protamine-based complexes, despite improving transfectability of mRNA, show lower transfection efficiency in comparison with liposome-based systems [22]. One of the possible reasons for this observation may lie in the strong hydrophilicity of Protamine, which makes it difficult to cross the cellular membrane. Another reason could be insufficient release of nucleic acid from the endosome into the cytoplasm, i.e., failure of endosomal escape. In fact, the majority of nanoparticle-based systems are internalized into cells though the endocytosis pathway [23]. Uptake through endocytosis involves internalization into an endocytic vesicle, fusion into the early endosomal compartment, maturation into a late endosome, and subsequent accumulation in the lysosome [24]. During maturation of the endosome, the pH decreases from physiological pH of 7.4, down to ~pH 6.5 in the early endosome, ~pH 6.0 in the late endosome, and ~pH 5.0 in the lysosome [25]. Several studies have indicated that escape from the endocytic pathway is the rate-determining step in the delivery of therapeutics, including nucleic acids, as failure to escape results in entrapment and potential degradation in the lysosome [26,27,28].

**Figure 2:**
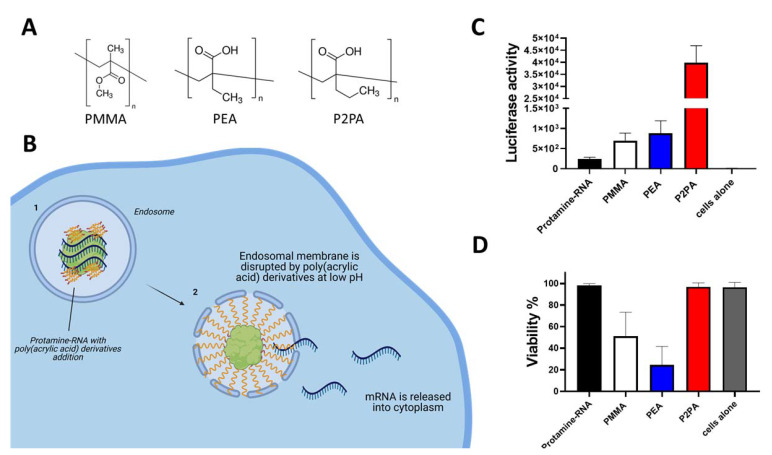
Improvement of Protamine-RNA complex transfectability by the addition of poly(acrylic acid) derivatives. (**A**) Chemical structures of used Endosome Destabilizing Agents (**B**) Schematic mechanism of action. At low pH PAA derivatives start to behave like surface active substance and destabilize the endosomal membrane allowing mRNA to escape degradation into the cytoplasm; (**C**) Addition of PAA derivatives (PMMA, PEA, P2PA) to Protamine-RNA (Luciferase) complexes improves transfection efficiency. All prepared particles contained 200 ng of Luciferase –coding mRNA; PMMA particles contained: 1.8 µg PMMA and Protamine 6 µg; PEA particles contained: 200 ng PEA and Protamine 1.2 µg; P2PA particles contained: 1.8 µg P2PA and 2 µg of Protamine. 100,000 HEK293 cells in RPMI complete medium were incubated for 24 h with indicated particles and after that time Luciferase activity was measured after addition of 25 µL of BrightGlo reagent (Promega); (**D**) Viability of HEK293 cells after 24 h exposure to Protamine-RNA-PAA complexes measured via LDH assay (Promega). Data represent triplicates mean value with SD.

To overcome the above-mentioned issue, it is possible to use endosome membrane-destabilizing agents in the formulations of Protamine-RNA. Poly(acrylic acid) (PAA) derivatives are polymers that show pH-dependent behavior. They are surface-active substances in acidic milieu, hence destabilizing biological membranes [29]. The endosomal low pH environment triggers destabilization of the membrane by PAA derivatives, which induces release of RNA from endosomes and its transfer to the cytosol (Figure 2B), allowing it to perform its biological activities [30].

We compared three different poly(acrylic acid) derivatives [29]: poly(2-ethyl acrylic acid) (PEA), poly(2-propyl acrylic acid) (P2PA), and polymethyl methacrylate-co-methacrylic acid (PMMA) (Figure 2A) by addition to Protamine-RNA formulations containing Luciferase-coding mRNA. We formulated different ratios of positive/negative charge particles and various ratios of endosome destabilizing agents (EDAs) within the negative part of complexes.

We observed that all investigated compounds helped transfection efficiency in HEK293 cells using Protamine-RNA complexes (Figure 2C). Among all tested formulations, polyplexes containing P2PA could improve the Luciferase signal by over 100 fold in comparison to Protamine-RNA complexes. Particles with addition of P2PA showed no toxicity while particles with PEA decreased cell viability (Figure 2D). Such results suggest that improvement of transfectability of RNA by Protamine can be obtained by simple addition of endosome destabilizing agents in the particles.

### 2.2. Protamine-Based Lipo-Polyplexes

**Figure 3:**
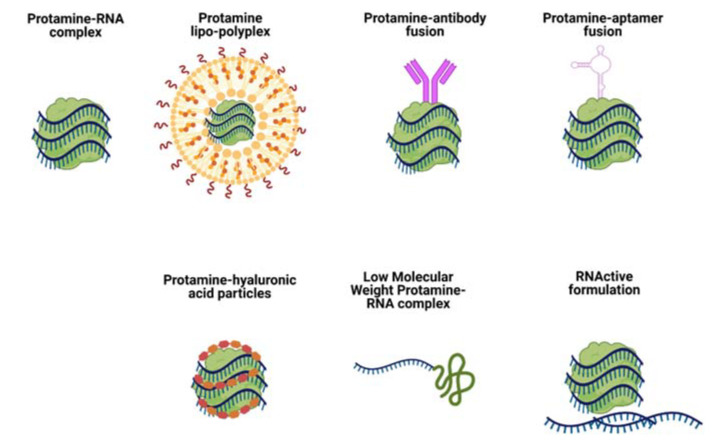
Schematic representation of Protamine-RNA formulations for RNA delivery mentioned in this review.

As a well-described profile protein with known safety, Protamine-based delivery systems can be modified in many ways to achieve various therapeutic outcomes. One of the most common strategies implemented for improvement of nucleic acid delivery via Protamine is its combination with lipids. Such formulations are called lipo-polyplexes, as they combine cationic biopolymers (i.e. Protamine) with lipids (cholesterol, DOTAP, etc.) (Figure 3, Protamine lipo-polyplexes).

Gao et al. observed in 1990s that the addition of poly(L-lysine) or Protamine radically reduced the particle size of the complex formed between DNA and DC-chol/DOPE cationic liposomes and improved DNA resistance to the nuclease activity [21]. The transfection efficiency of resulting lipo-polyplexes was also significantly improved than that of corresponding liposome/DNA complexes. In follow-up studies, L. Huang with colleagues established that addition of Protamine sulfate, USP, as a condensation agent was superior to poly(L-lysine) and other types of Protamines, in the context of condensing DNA in cationic lipo-polyplexes [19,31].

The effect of the combination of both DOTAP and Protamine proved to be beneficial for DNA delivery in vitro and in vivo. Aragoa et al. tested multiple conditions of protamine-DOTAP poly-lipoplexes in their ability of direct liver delivery and transfection [32]. Protamine here was used as a complexation agent to decrease the size of prepared formulations and as a transfection enhancer. Addition of Protamine indeed decreased the size of investigated particles from 302 to 181 nm [32]. Such observations were important in the context of transfection optimization, as small particles seem to be more readily endocytosed by the cells [33]. The optimal amount of Protamine in the investigated complexes was established as 0.4 μg per 1 μg of DNA (with an overall positive/negative ratio of polyplex being 4:1). This study was also the first efficacy demonstration of the combination of the condensing action of Protamine and targeting effect of the ligand (Asiolofetuin, AF, which is an asioglycoprotein receptor ligand, used in liver-targeting) in vivo [34,35]. Mice injected intravenously with Protamine-DOTAP-AF lipo-polyplexes containing Luciferase-coding DNA increased target gene expression in the liver compared to plain AF-DOTAP lipoplexes, with no observed toxicity.

In recent studies, Siewert et al. investigated the change in structural properties of Protamine-lipid (DOTAP) nanoparticles that would potentially lead to improved transfection efficacy [36]. Lipid nanoparticles comprising mRNA were manufactured at various DOTAP:Protamine ratios, using different assembly routes, to obtain nanoparticles with an optimized core–shell organization. The authors suggested that addition of protamine to negatively charged DOTAP/RNA pre-complexes affected the pre-existing lipoplexes’ organization and the resulting structure was more intricate than a plain core–shell geometry. Different structural morphologies corresponded with different transfection efficacies with the strongest improvement in biological activity (transfection of tumor cells in vitro) observed in particles of direct DOTAP/Protamine mixture added on mRNA, with a composite organization of the lamellar patches inside the unstructured matrix. The authors suggested it might have been due to facilitated mRNA release from such nanoparticles resulting from the self-assembly of complexing agents.

## 3. Immunostimulation by Protamine-RNA Formulations: Towards RNA Vaccines

Inducing immunity via nucleic acid-based vaccines is a fast growing and promising branch of medicine. RNA is especially promising due to its natural property as a danger signal that allows it to stimulate adaptive immune responses, hence in vaccine development RNA can act as an adjuvant [37,38].

### 3.1. Adjuvant and Immunostimulatory Properties of Protamine-RNA Formulations 

The first reports of using Protamine as an mRNA condensation and protection agent for vaccination was published in 2000 by Hoerr et al. [3]. The authors proved that mice injected with Protamine-protected mRNA coding for the model antigen of beta-galactosidase (βgalZβgα_n_ RNA) were able to produce antigen-specific cytotoxic T lymphocytes (CTLs) and IgG antibodies against this antigen. Interestingly, the specific immune response was detectable only after injection in ear pinnae and not after intravenous injections. Only 1 μg of Protamine-condensed βgalZβgα_n_ RNA was sufficient for in vivo CTL priming. It was then reported for the first time that RNA can be qualified as a danger signal since when stabilized (modified or mixed with Protamine) it triggers innate immunity [37]. Indeed, it was thereafter found that RNA stimulates endosomal-resident Toll-like receptors 7 and 8 (TLR 7 and 8) [7,38]. When triggered, TLRs induce specific intracellular activation pathways that can result in the expression of different types of innate immune response molecules, such as type I interferons and TNF-alpha (Figure 1B) [10]. Unmodified single-stranded RNA (ssRNA) is recognized by human TLR7 (expressed in plasmacytoid dendritic cells) and human TLR8 (expressed in monocytes). A TLR-induced cellular response consists of the activation of different signal transduction cascades and ultimately leads to induction of secretion of cytokines (e.g., IL-12, IFNα, TNFα) [37,39].

This feature suggested the possibility of using RNA as an anti-tumor treatment [7]. Glioblastoma-challenged mice were treated with series of intra-tumoral injections consisting of naked mRNA, CpG DNA, mRNA condensed with Protamine or Protamine alone. Injections of mRNA alone or Protamine-protected mRNA as well as injections of CpG DNA into tumors led to a significant delay in tumor growth and in the long term, circa 20% of mice remained tumor-free in all nucleic acid-injected groups. The tumor-free mice were subsequently re-challenged with glioblastoma cells. None of the mice that had recovered from the primary tumor graft as a consequence of nucleic acid treatment showed any palpable tumors, which indicated that immunotherapy of solid tumors using RNA as a danger signal led to long-term anti-tumor immunity. It was postulated that Protamine-stabilized RNA could represent a safer alternative replacement of CpG DNA-based adjuvants to be applied in the context of many immunotherapeutic or prophylactic treatments.

Fotin-Mleczek and colleagues explored another aspect of immunostimulatory RNA formulations for cancer immunotherapy [40]. Since mRNA complexation with Protamine can inhibit translation of mRNA, the authors investigated a new formulation consisting of two components: mRNA complexed with Protamine for providing good innate immune stimulation, and free mRNA for antigen expression. This was named RNActive vaccine (Figure 3, RNActive formulation) [41]. Animal studies showed a delay in tumor growth (melanoma cell line B16 expressing ovalbumin) of about 10 days in groups vaccinated with a two-component formulation containing OVA-coding mRNA. The authors observed significant superiority of the two-component vaccine compared with a single component (naked mRNA). This two-component vaccine induced complete adaptive immune responses, including activation of antigen-specific B and T cells. The study was repeated with mRNA coding a weaker antigen, PSMA (Prostate carcinoma-associated antigen) and gave lower, but detectable levels of innate and adaptive immune responses. The two-component RNActive formulation was also effective as a therapeutic vaccine: mice receiving the two-component OVA vaccine after tumor transplantation displayed inhibited tumor growth rates in comparison with non-vaccinated mice.

### 3.2. Clinical Trials with Protamine as an mRNA Carrier

Protamine has been widely used in clinics as a heparin antagonist and in slow-release insulin formulations for many years. When it comes to its application as an RNA carrier, there have been several clinical trials performed in the past 20 years aiming at testing Protamine-RNA complexes’ performance in cancer immunotherapy in patients. All of the below mentioned trials aimed at assessment of safety and efficacy. In all described studies, vaccines were well tolerated, with most common side effects being skin irritation at injection sites and flu-like symptoms. Every investigated Protamine-mRNA based vaccine induced detectable levels of appropriate immune responses, however, the results suggested the necessity for further optimization and the potential need, in the context of cancer, to combine the system with checkpoint inhibitors or other anti-cancer therapies, such as local radiotherapy. Published studies are described in detail in the below section and summarized in Table 1.

Just after the evaluation of naked mRNA vaccine in melanoma patients [48], the Tuebingen-based research group explored Protamine-mRNA complexes in a Phase I/II vaccination trial in metastatic melanoma patients (NCT00204607) [42]. In this study, 21 patients with metastatic melanoma were injected with Protamine-condensed mRNAs coding for melanoma antigens. The most frequently occurring side effect was an inflammatory skin reaction at the injection site. Fatigue was reported in 86% of the patients. No adverse effects exceeding grade 2 were observed. The addition of Protamine caused more intensive injection site reactions compared with naked mRNA [42]. A reproducible increase of vaccine-induced T cells was observed in two out of four immunologically evaluable patients. One of seven patients with measurable disease showed a response of lung metastases at the end of the treatment. Upon ongoing vaccinations these lesions regressed completely 13 months after starting the therapy. The authors concluded that although Protamine-protected mRNA is feasible and safe as a vaccination method, the clinical or immunological responses were low, probably due to cellular immunosuppression (significantly decreased levels of Foxp3+/CD4+ regulatory T cells in treated patients). Indeed, in some murine models, Protamine-RNA based immunotherapies combined with low doses of anti-CTLA-4 or anti-PD-1 showed synergistic effects, resulting in complete tumor rejection [49].

The RNActive technology was tested in healthy volunteers using mRNA coding for a rabies virus glycoprotein (NCT02241135) [45]. Healthy adults received three doses of mRNA and Protamine containing vaccines (CV7201) intradermally or intramuscularly, with a booster after one year. The goals were to assess safety and tolerability as well as to determine the lowest dose of the vaccine needed to elicit rabies virus neutralizing titers. Rabies virus was selected as a model antigen to explore mRNA technology in humans, as the population is naïve to the virus unless previously vaccinated. This vaccination was also proven safe and well tolerated. All described adverse reactions were transient and mild to moderate in severity. There were four serious adverse events: one due to human error and a case of Bell’s palsy, nasal septum deviation and campylobacter infection.

Analysis of functional antibody titers against the rabies virus revealed clear differences between administration with needle-syringe or needle-free injector devices. In needle-syringe cohorts there were no detectable levels of antibody responses, while 77% of the group vaccinated with the injector device developed detectable virus neutralizing titers. This pattern was observed in both intramuscular and intradermal vaccine administration. In most patients, RABV-G-specific IgM titers peaked at day 21, IgG peaked at day 42. One year after the boost there was no change in RABV-G specific IgM antibody levels. This predominantly IgG response is indicative of an established immune memory response during the initial vaccination schedule. RAVB-G-specific CD4+ T cells were increased at day 42 compared to baseline, they declined to baseline at day 91.

RNActive vaccines were also evaluated in cancer patients [43,46]. Vaccine against prostate cancer, CV9103, that contained four different mRNAs and Protamine, was administered intradermal to 44 patients at up to 1280 micrograms RNA per injection. Side effects included local reactogenicity and fatigue, pyrexia, chills and influenza-like illness. Immune responses were detected in the majority of the patients (and those survived also longer than immunological non-responders). One patient demonstrated a PSA response [43]. Follow-up studies with a vaccine (CV9104) including two more mRNA species (coding for two additional antigens) have been performed. However in a placebo control study with 197 patients, there was no impact of the CV9104 vaccine on overall survival or progression free survival [44]. 

The CV9201 vaccine encoding five non-small lung cancer antigens was tested in 46 patients in a I/IIa dose-escalation trial. The objectives of the study were safety assessment and evaluation of T cell responses against the five antigens. Different doses were investigated, ranging from 400 to 1600 μg of RNA per intradermal injection. Most of the adverse effects were mild-to-moderate injection site reactions and flu-like symptoms, whereas three patients had grade 3 related adverse events. In the phase IIa trial, antigen-specific immune responses against more than one antigen were detected in 63% of patients. No clear dose–response relationship was observed, but higher frequencies of immune responses in patients treated with lower mRNA doses were noticed. Nine patients had stable disease as best overall response in 29 evaluated patients. Median overall survival was 11.5 months in the total population. No cases of clinically apparent autoimmune disease were observed. In part IIa of the trial, patients were injected with 1600 μg of CV9201. Both cellular and humoral immune responses were detected against all antigens, however the responses were modest and revealed high inter-patient variability. No objective tumor responses were observed with the vaccine and, again, they were associated with tumor-induced inhibition of the immune system. According to the authors, the vaccine showed an acceptable tolerability profile and evidence of immune activation. In a follow-up Phase Ib study, CureVac evaluated combined therapy consisting of RNActive CV9202 encoding six non-small cell lung cancer-associated antigens and local radiotherapy for the treatment of stage IV non-small cell lunch cancer, NSCLC (vaccine called BI1361849). Again, the most common side effect was an injection site reaction and flu-like symptoms. Three patients had grade 3 adverse events like fatigue and pyrexia. In comparison with baseline, immunomonitoring studies revealed vaccine-induced antigen-specific immune responses in 84% of patients. Antigen-specific antibody levels were increased in 80%, and functional T cells in 40% of the patients. Frequencies of functional CD4+ and CD8+ T cells following BI1361849 combined with radiotherapy increased over time. One patient achieved a partial response with decreasing measurable tumor size. Twelve out of 26 patients demonstrated stable disease as best overall response. The immunomonitoring results were comparable to those observed with CV9201 vaccine alone without radiation [46]. An increase in tumor antigen-specific T cells and antibodies were detected in all experimental groups. Again, the observed responses were at low frequencies of CD4 and CD8 cells. The group suggested for both vaccine studies to be tried in combination with immune checkpoint inhibitors to help break the tolerance against endogenous antigens, e.g., by enhancing effector T-cell function and inhibition of Tregs [50].

## 4. Protamine-siRNA Targeted Delivery

RNA interference (RNAi) describes the fundamental process in eukaryotes in which double-stranded RNA (dsRNA) induce cleavage of mRNA with complementary sequences [51]. Mechanisms of gene silencing via RNA interference is a promising treatment strategy in several diseases such as cancer, genetic disorders, autoimmune diseases or viral infections [52,53]. However, as in case of mRNA, the main difficulty in clinical applications of siRNA is its delivery to the cytoplasm [54]. Since siRNA is used for specific gene silencing, it also is necessary to deliver it directly to the target tissue or cells. That is why most of the approaches for siRNA carrier design focus on its precise targeting.

Song et al. were the first group to use Protamine to deliver siRNA via an antibody Fab fragment fused with Protamine (Figure 3, Protamine-antibody fusion) [55]. The Fab antibody fragment (F105P) directed against the HIV-1 envelope fused to Protamine was used to deliver siRNA and silence gene expression specifically in HIV-infected cells or cells transfected to express HIV envelope glycoprotein gp160 (*env*). siRNA was bound to that fusion protein through ionic interactions, without any need of covalent coupling. Delivery was specific to *env*-bearing cells both in vitro and in vivo and systemic distribution was possible by conventional intravenous administration. The group managed to introduce siRNA into difficult-to-transfect CD4+ T cells and suppress HIV production in already infected cells. It was proven in the study that the Fab-Protamine fusion protein can be modified by replacing the Fab fragment with a single-chain antibody or a cell surface receptor ligand. The authors argued that Protamine-siRNA complexes are unlikely to form nanoparticles that could be potentially trapped by reticuloendothelial cells.

The group of Song also evaluated siRNA-targeting antibody fusion in breast cancer treatment [56,57]. They used *Polo-like kinase 1* (*PLK1*)-specific siRNA complexed with a Her2-ScFv-Protamine fusion protein (5F-P). In vitro, this complex could suppress Her2+ breast cancer cell lines and primary human cancer cells by targeted gene expression inhibition, reducing proliferation and increasing apoptosis of Her2+ breast cancer cell lines. In vivo, complexed siRNA administered intravenously could suppress *PKL1* gene expression in tumor and thus trigger tumor cell apoptosis. That resulted in a delay of tumor growth and reduced metastasis.

Song’s strategy to fuse Protamine to a targeting agent was also used by Lieberman, Shimaoka and colleagues [58]. The authors showed that targeting the human integrin lymphocyte function-associated antigen-1 (LFA-1) allowed efficient delivery of siRNAs and cell type-specific gene silencing in primary lymphocytes, monocytes and dendritic cells. To achieve specific gene silencing only in activated leukocytes, the authors constructed a Protamine fusion protein from a scFv that preferentially recognized activation-dependent conformational changes in LFA-1 [59].

Another method to generate siRNA-Protamine-antibody complexes was described by Baumer et al. [60]. The procedure consisted of conjugating the protamine N- terminus to a sulfo-sMCC cross-linker allowing coupling via cysteine residues to the IgG backbone. The formed Protamine-Ab molecule could then be mixed with siRNA to generate siRNA-Protamine-antibody complexes. This coupling method gave similar results to genetic fusion.

Another reported strategy to target siRNA delivery with the use of Protamine is based on the use of aptamers as targeting moieties (Figure 3, Protamine-aptamer fusion). Such a method was proposed by Gong et al. [61]. The construct consisted of an ErbB3 aptamer, Protamine and siRNA and the particles were called APRs.

An aptamer is a DNA or RNA oligonucleotide that recognizes and binds to a targeted protein with high affinity and specificity [62]. In the proposed nanoparticle design, Protamine acted as a bridge between the aptamer and siRNA. In these experiments, the aptamer against ErbB3 was the targeting agent for breast cancer cells, and the siRNA was directed to oncogene survivin. Both aptamer and siRNA were 2′OMe modified to prevent their degradation. Particles generated by mixing aptamer-Protamine-siRNA were smaller than 100 nm in diameter and were proven to have high affinity and specificity to target breast cancer cells expressing ErbB3 (HER3). In in vivo studies, APR particles could silence survivin expression and induce cell apoptosis and inhibition of proliferation. APR particle administration could inhibit tumor growth in tumor-bearing nude mice. No toxicity of the treatment was observed.

A similar approach of aptamer-binding to Protamine to deliver target nucleic acids was also employed by the group of Zu in DNA and siRNA-based treatment of anaplastic large cell lymphoma (ALCL) [63]. Additionally, in this case, aptamer-Protamine-nucleic acid particles bound specifically to lymphoma cells and could efficiently kill targeted cells. Functional studies performed by the authors demonstrated that, combining a cell-selective chemotherapy using a drug payload and oncogene-specific gene therapy using siRNAs, resulted in particles that could effectively kill lymphoma cells with little toxicity to off-target cells.

A different approach in tumor targeting was proposed by Wu and Wang [64]. They mixed Protamine, miRNA and Hyaluronic Acid (HA) to obtain tumor-targeted particles for triple-negative breast cancer therapy (Figure 3, Protamine-hyaluronic acid particles). HA was used to target delivery because of the specific binding ability of HA to CD44 molecules, which are overexpressed in a variety of tumor tissues [65,66,67,68]. Nanocapsules were composed of cationic Protamine sulfate and negatively charged HA+RNA by self-assembly due to negative-positive charge interactions between HA+RNA (negative charge) and Protamine (positive charge). The particles could efficiently target triple-negative breast cancer cells and deliver miR-34a, which triggered their apoptosis. The anticancer effect was confirmed in in vivo studies of breast tumor-bearing mice. Administration of particles suppressed tumor growth and induced tumor cell apoptosis through targeting CD44 and the Notch-1 signaling pathway.

Another very interesting strategy was employed by the group of Park and Choi [69]. They digested Protamine with thermolysine to obtain Low Molecular Weight Protamine (LMWP), which proved to be a cell penetrating peptide [70] that they subsequently used to covalently complex and deliver siRNA (Figure 3, Low Molecular Weight Protamine RNA complex). The authors compared LMWP with TAT peptide in its ability to deliver siRNA into tumor cells in vitro and in vivo. siRNA could be delivered to tumors by LMWP-mediated systemic injection without causing inflammatory side effects. The group’s research confirmed that LMWP possesses significantly reduced antigenicity, mutagenicity and complement-activating activity in comparison with its parent Protamine molecule. The LMWP-siRNA complexes were approximately 50 nm and showed a prolonged circulation time in mouse models providing high fluorescence in tumors. Mice treated with VEGF-targeted siRNA-LMWP complexes showed significant tumor regression.

## 5. Conclusions

Protamine is a very flexible and versatile compound that has a broad range of applications in research and medicine. Due to its clinical safety (although side effects such as rare anaphylactic response and contraindications such as fish allergies must be taken into account [71]), it is eagerly applied to many areas of drug delivery research. The most exploited feature of this small protein is its high positive charge due to an arginine-rich sequence, as the positively charged Protamine can spontaneously assemble with any negatively charged substance, including DNA, RNA or heparin.

Protamine was identified very early on as a compound that can enhance transfectability in vitro with the first experiments of this matter being done 60 years ago. It has no detectable cytotoxicity in vitro up to a concentration of 10 mM (over 40 g/L), whereas other transfection reagents such as polyethylenimine showed significant toxicity above a concentration of 5.0 mM. In the last twenty years, Protamine was used to protect and deliver different forms of RNA (mRNA, immunostimulating RNA or siRNA) in order to generate vaccines and anti-cancer drugs. It is expected that Protamine-containing RNA drugs will be approved in the near future and that thanks to the versatility and safety of this compound, new superlative RNA formulations will be created in order to generate efficient new drugs and vaccines.

## Figures and Tables

**Table 1:** Published clinical trials with Protamine-mRNA.

Condition	Protamine Formulation	Number	Reference
Metastatic Melanoma	Protamine ICM	NCT00204607	[42]
Prostate Cancer	RNactive CV9103	EudraCT 2008-003967-37	[43]
Prostate Cancer	RNactive CV9104	NCT01817738	[44]
Rabies	RNactive CV7201	NCT02241135	[45]
Non-small Cell Lung Cancer	RNactive CV9201	NCT00923312	[46]
Non-small Cell Lung Cancer	RNactive CV9202	NCT01915524	[47]
